# Nucleophilic Water Capture or Proton Loss: Single Amino Acid Switch Converts δ‐Cadinene Synthase into Germacradien‐4‐ol Synthase

**DOI:** 10.1002/cbic.201700531

**Published:** 2017-11-23

**Authors:** Marianna Loizzi, Veronica González, David J. Miller, Rudolf K. Allemann

**Affiliations:** ^1^ School of Chemistry Cardiff University Main Building, Park Place Cardiff CF10 3AT UK

**Keywords:** cations, enzymes, natural products, reaction mechanisms, site-directed mutagenesis

## Abstract

δ‐Cadinene synthase is a sesquiterpene cyclase that utilises the universal achiral precursor farnesyl diphosphate (FDP) to generate predominantly the bicyclic sesquiterpene δ‐cadinene and about 2 % germacradien‐4‐ol, which is also generated from FDP by the cyclase germacradien‐4‐ol synthase. Herein, the mechanism by which sesquiterpene synthases discriminate between deprotonation and reaction with a nucleophilic water molecule was investigated by site‐directed mutagenesis of δ‐cadinene synthase. If W279 in δ‐cadinene synthase was replaced with various smaller amino acids, the ratio of alcohol versus hydrocarbon product was directly proportional to the van der Waals volume of the amino acid side chain. DCS‐W279A is a catalytically highly efficient germacradien‐4‐ol synthase (*k*_cat_/*K*_M_=1.4×10^−3^ μm s^−1^) that produces predominantly germacradien‐4‐ol in addition to 11 % δ‐cadinene. Water capture is not achieved through strategic positioning of a water molecule in the active site, but through a coordinated series of loop movements that allow bulk water access to the final carbocation in the active site prior to product release.

## Introduction

Present in all kingdoms of life, terpene synthases catalyse highly complex biosynthetic reactions, in which achiral, linear isoprenyl diphosphates are converted into complex often cyclic or polycyclic structures.^1^, ^2^ In most cases, these carbocationic reaction cascades are characterised by high stereo‐ and regioselectivity and involve changes in the hybridisation of up to half of the carbon atoms. The final carbocation can either lose a proton to generate a hydrocarbon product or react with water to produce a terpene alcohol.^1^ Subsequent biosynthetic reaction steps convert the products generated by terpene synthases into tens of thousands of terpenoids that act, among other things, as pigments; phytoalexins; semiochemicals or in primary metabolism as sterols, carotenoids and ubiquinones. Terpenoids have many important applications, for instance, as drugs, fragrances, pesticides, fuels or as food additives.^1^, ^2^

Examination of the structures of terpene synthases and the mechanisms of the catalysed reactions has revealed common structural features and distinct phases of activity.^1^, ^2^, ^3^, ^4^, ^5^, ^6^ Class I terpene synthases share a predominantly α‐helical fold with an active site lined with mostly hydrophobic and aromatic amino acid residues; they contain two conserved Mg^2+^‐binding motifs (DDXXD and NSE/DTE) on opposite sides of the active site.^2^ They provide a three‐dimensional template to bind the flexible substrate and chaperone the carbocationic intermediates along distinct reaction paths. Class I terpene synthases initiate the chemical reaction by catalysing the cleavage of the carbon–oxygen bond of the substrate to generate a tightly bound diphosphate (PP_i_)–carbocation pair.^2^, ^7^ Single‐crystal X‐ray structures of several sesquiterpene synthases complexed with (*E*,*E*)‐farnesyl diphosphate (FDP, **1**), several analogues of **1**, Mg^2+^ and PP_i_, together with molecular dynamics simulations, have provided strong support that loop movements and conformational changes are required to form the closed form of the enzyme, in which substrate **1** is in a reaction‐ready conformation.^2^, ^3^, ^4^, ^5^, ^6^, ^7^, ^8^ After conformational rearrangements of enzyme and substrate necessary to form the Michaelis complex, the chemical reaction occurs, with major contributions from carbocation stabilisation by the π electrons of aromatic amino acid residues, PP_i_ carbocation interactions and general acid–base catalysis by PP_i_ and/or the enzyme.^1^, ^2^, ^3^, ^4^, ^5^, ^6^, ^7^, ^8^, ^9^, ^10^, ^11^, ^12^ The closed conformation of sesquiterpene synthases that generates hydrocarbon products also prevents access of bulk solvent, which avoids quenching of the reactive carbocationic reaction intermediates by water.^1^, ^2^, ^3^, ^6^, ^7^, ^8^, ^9^, ^10^, ^11^

**Scheme 1:**
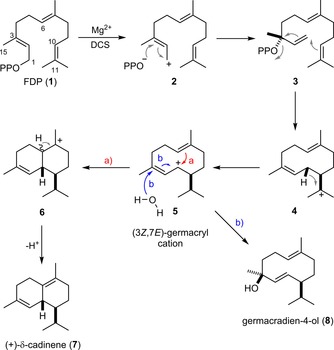
Catalytic mechanisms of the DCS (pathway a) and GdolS (pathway b) catalysed conversions of **1** to **7** and germacradien‐4‐ol (**8**).

In contrast to the wealth of knowledge available on the mechanistic details of sesquiterpene synthase catalysed reactions, in which the last cationic intermediate is deprotonated by PP_i_, little information is available for terpene alcohol synthases, such as epicubenol,^13^ hedycaryol,^14^ avermitilol,^15^ epicedrol^16^ and germacradien‐4‐ol synthases^17^ (GdolS), which generate their products through the reaction of the final carbocation with water. The mechanism by which sesquiterpene synthases discriminate between deprotonation or water capture has not been explored in detail. In enzymes that generate alcohols, deprotonation of the final cation must be prevented, and capture of the final carbocationic intermediate must be tightly controlled to prevent quenching of early cationic intermediates, whereas synthases that generate terpene alcohols must prevent deprotonation of the final carbocationic intermediate. Clearly, terpene synthases have evolved to control water access and reactivity. Tightly bound water molecules can be found in the active sites of terpene synthases in their closed conformation, even for synthases that do not generate alcohol products.^2^, ^7^, ^9^ This finding might suggest that these sequestered water molecules could be responsible for nucleophilic capture of the final carbocationic species.^2^, ^14^ However, a previously published investigation into the structure and mechanism of GdolS revealed that the reaction of the final carbocation most likely depended on specific loop movements of the enzyme that allowed bulk water to access the active site.^18^ GdolS must prevent deprotonation from C6 in intermediate **6**, so as not to produce δ‐cadinene (**7**; Scheme 1), which in contrast is the pathway for catalysis by δ‐cadinene synthase (DCS).^9^

In selina‐4(15),7(11)‐diene synthase from *Streptomyces pristinaespiralis*, a G1/2 helix break motif, combined with a diphosphate sensor–linker–effector motif that is conserved throughout bacterial sesquiterpene synthases, has been proposed to play a major role in substrate binding and active‐site closure.^19^ This “kink” in the G‐helix has also been noted as a potentially important catalytic feature in human squalene synthase^20^ and hedycaryol synthase.^14^ Interestingly, DCS was converted into a GdolS through saturation mutagenesis by Keasling and co‐workers.^21^ A re‐evaluation of that work reveals that the amino acid residues that generate this change of function are located in the G1/2 helix break motif (see below).

DCS from *Gossypium arboreum* produces **7** in the first committed step of the biosynthetic pathway to the phytoalexin gossypol (Scheme 1).^22^ Despite little sequence identity outside the conserved Mg^2+^‐binding motifs, DCS and GdolS share the typical structure of class I terpene synthases in the catalytic domain;^9^, ^18^ many aspects of their active‐site compositions and the respective catalytic reaction pathways from **1** to **7** or **8** are shared (Scheme 1). The co‐crystal structure of DCS, Mg^2+^ and the substrate analogue (2*Z*,6*E*)‐2F‐farnesyl diphosphate (2F‐FDP) revealed an unusual Mg^2+^‐binding motif, in which the NSE/DTE motif is replaced by a second DDXXD motif.^9^ Interestingly, depending upon the substrate used, DCS appeared to follow a 1,6; 1,10 or 1,11 ring closure;^10^ this suggested that these pathways were energetically similar and that DCS might have some inherent promiscuity, despite its high fidelity when acting on **1**.^10^ Only the N‐terminal tail of the N‐terminal β domain of DCS is involved in catalysis and plays an important role in protecting the active site from water. Truncated proteins that lack the first 8 and 20 amino acids of the β domain produce increasing amounts of **8**.^23^

The double‐mutant protein DCS‐N403P/L405H was shown to convert **1** into **8** (93 %) and an additional unidentified cyclic sesquiterpene alcohol.^21^ However, the catalytic activity of DCS‐N403P/L405H is severely compromised relative to that of the wild‐type (WT) enzyme.

**Figure 1:**
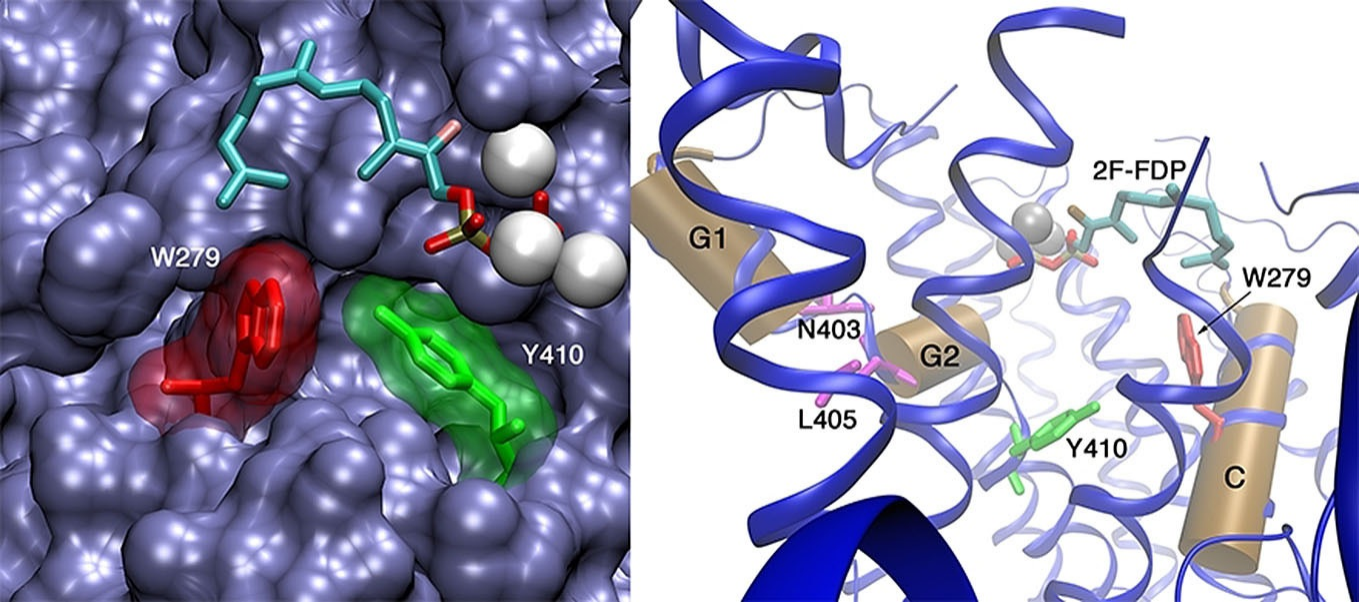
Left: view of the active‐site cleft of DCS, showing the bulk surface of the enzyme in blue. Mg^2+^ ions are depicted as silver spheres; Y410 and W279 are shown with their van der Waals radii in green and red, respectively. Right: sketch of the active site of DCS; N403 and L405 are at the hinge points of the G1/G2 helix break (magenta). Y410 is shown in green and W279 in red; helices G1, G2 and C as tan cylinders; and Mg^2+^ ions as silver spheres. 2F‐FDP is shown as bonds, but in this crystal structure its hydrocarbon tail did not bind within the cleft (PDB ID: 3G4F^9^).

In DCS‐N403P/L405H, the active site is exposed to solvent through a potential water channel created by alteration of the G‐helix residues.^21^ Analysis of the X‐ray structure of DCS reveals that the aromatic residues W279 and Y410 are closer to the isoprenyl chain of the substrate and on the opposite side of the active‐site contour relative to N403 and L405. W279 is on the C helix of DCS and within 7 Å of Y410, just below the G2 helix and towards the bottom of the active‐site cleft (Figure 1). These two residues are ideally placed not only to stabilise carbocationic intermediates during the formation of **7**, but also to form hydrophobic interactions that may help to control the active‐site conformation of **1** and mediate active‐site closure and opening. Hence, to test the hypothesis that alteration of W279 can disrupt hydrophobic interactions with Y410 and **1** and allow increased water access to the active site, the contribution of W279 to catalysis was explored by sitedirected mutagenesis. Herein, we show that single amino acid changes can convert DCS into GdolS that produce up to 90 % **8** with high catalytic efficiency. The results suggest that W279 plays a key role in shielding the active site of DCS from solvent.

## Results and Discussion

DCS‐His_6_ was produced in *Escherichia coli* and its catalytic properties determined. Steady‐state kinetic experiments with radiolabelled [1‐^3^H]**1** 9, 10 revealed a turnover number, *k*_cat_, of 1.26×10^−3^ s^−1^; a Michaelis constant, *K*_M_, of 0.58 μm (Table 1) and a catalytic efficiency, *k*_cat_/*K*_M_, of (2.17±0.4)×10^−4^ s^−1^ μm ^−1^, which was identical to the value previously measured for WT‐DCS with no His‐tag (*k*_cat_/*K*_M_=(3.1±0.2)×10^−3^ s^−1^ μm ^−1^).^9^

**Table 1:** Kinetic data and products generated from **1** with DCS and DCS‐W279 mutants.

Enzyme	*k*_cat_	*K*_M_	**3 a**	**4**	*k*_cat_/*K*_M_
	[×10^−13^ s^−1^]	[μm]	[%]^[a]^	[%]^[a]^	[×10^−3^ μm ^−1^ s^−1^]
WT‐DCS^11^	1.00±0.4	3.20±0.02	98	2	3.1±0.2
DCS‐His_6_	1.27±0.005	0.58±0.11	90	10	2.17±0.4
DCS‐W279E	0.59±0.01	1.45±0.09	50	50	0.41±0.04
DCS‐W279Q	3.00±0.02	8.00±4.00	60	40	0.25±0.13
DCS‐W279D	0.67±0.01	9.24±3.00	40	60	0.07±0.02
DCS‐W279L	3.80±0.09	9.45±1.10	68	32	0.40±0.05
DCS‐W279M	1.09±0.04	1.90±0.20	65	35	0.57±0.06
DCS‐W279A	3.12±0.01	2.23±0.51	11	89	1.40±0.32
DCS‐W279Y	0.73±0.01	4.28±0.70	83	17	0.17±0.01

[a] Percentage of total products.

Analysis of the pentane‐extractable products arising from incubations with **1** by GC‐MS showed that, in addition to **7**, 11 % of **8** was produced by the His‐tagged enzyme. The products were identified by comparison with the GC retention times and mass spectra of authentic products generated through the incubation of **1** with WT‐DCS and GdolS from *Streptomyces citricolor*.^18^

To address the role of W279 during DCS catalysis, tryptophan was replaced by Glu, Gln, Asp, Leu, Met, Als and Tyr and the pentane‐extractable products generated from **1** were analysed by GC‐MS. Remarkably, DCS‐His_6_‐W279A produced only 11 % **7** and 81 % **8** (Table 1); a product ratio that represents an almost complete reversal relative to that measured for DCS‐His_6_. The values of *k*_cat_ (3.12×10^−3^ s^−1^) and *K*_M_ (2.23 μm) were similar to the values measured for DCS‐His_6_; this indicated that replacement of the hydrophobic and bulky indole group with hydrogen allowed water access to the active site to efficiently quench the intermediate (3*Z*,7*E*)‐germacryl cation (**5**) without loss of the catalytic efficiency. If W279 was replaced by tyrosine, the quantity of alcohol **8** formed was only slightly increased relative to that of DCS‐His_6_.

**Figure 2:**
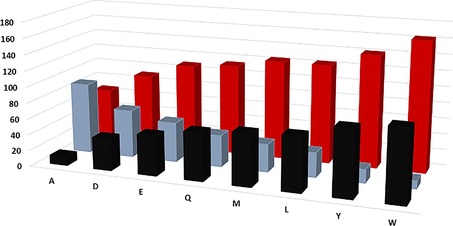
Histogram of the percentage distributions of products **7** (▪) and **8** (▪) generated by DCS‐His_6_ and its mutants versus the van der Waals volume^25^ of residue 279 (▪).

GC‐MS analysis of the pentane‐extractable products generated by DCS‐His_6_‐W279M and DCS‐His_6_‐W279L, in which residues with similar hydrophobicity, but with reduced van der Waals volume, replaced tryptophan,^25^ showed increased amounts of alcohol relative to that of DCS‐His_6_ (**7** and **8** in approximately 2:1 ratio). These results show that the relative amounts of **7** and **8** are dependent on the volume of the side chain of residue 279. The values of *K*_M_ for DCS‐His_6_‐W279M and DCS‐His_6_‐W279L were slightly increased, relative to that of the DCS‐His_6_ (Table 1). Smaller residues that were hydrophilic, rather than hydrophobic, were tested to examine the possibility that hydrophilic residues might form a repulsive interaction with Y410, leading to a poorly defined active site that compromised the catalytic activity. Alternatively, an increase in the mobility of the G1/G2 helix break motif might generate larger quantities of **8**. Consequently, W279 was replaced with glutamine, glutamate and aspartate. When incubated with **1**, DCS‐His_6_‐W279Q, DCS‐His_6_‐W279E and DCS‐His_6_‐W279D generated decreasing amounts of **7** and an increasing proportion of **8**, with the ratio of the two products showing a near**‐**linear relationship between the van der Waals volume of the amino acid and alcohol production (Figure 2 and Table 1). This provides powerful evidence that the van der Waals volume of the C‐loop residue 279 is of central importance for product distribution in DCS catalysis.

**Figure 3:**
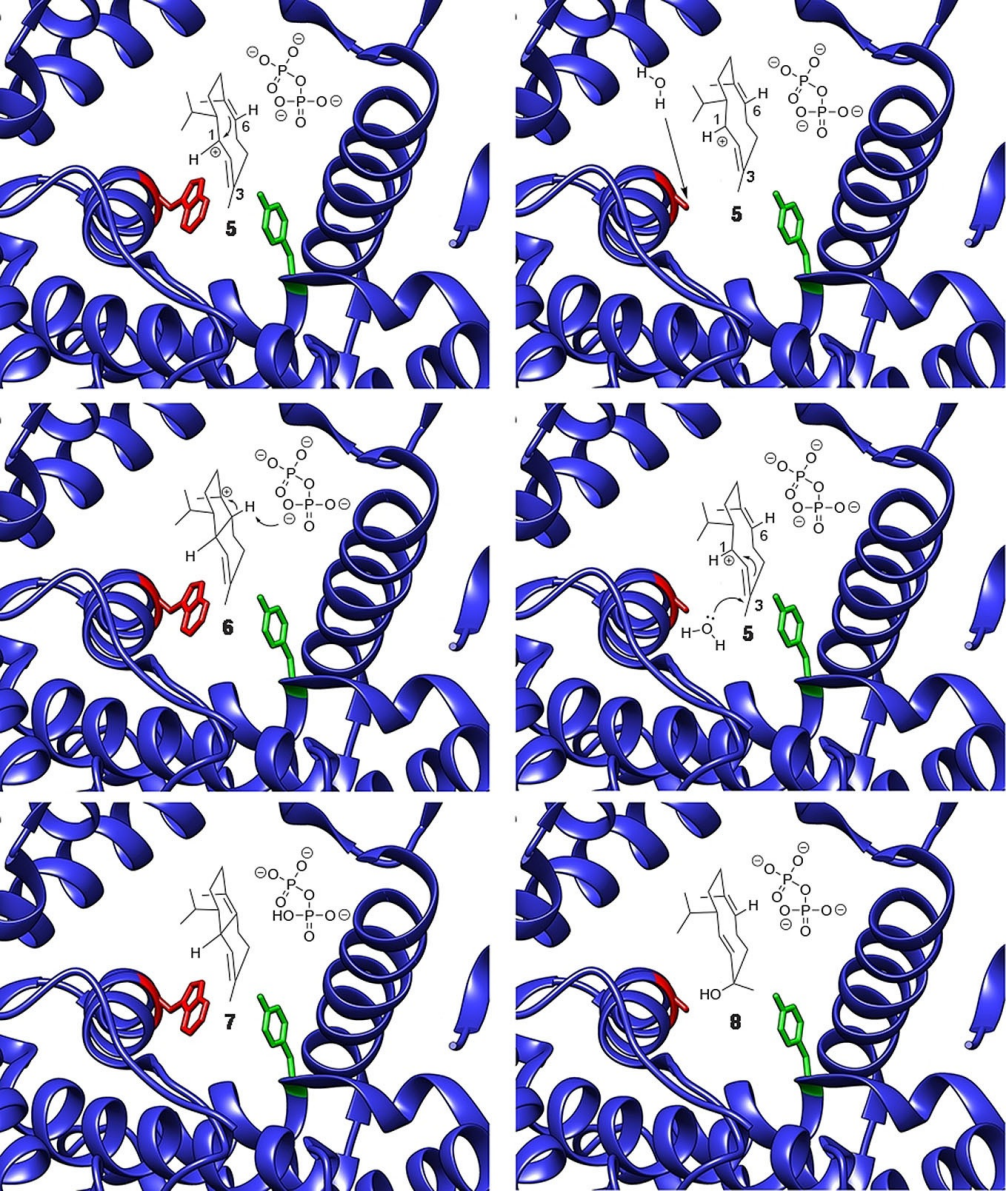
Representations of the active site of WT DCS (left)^9^ and DCS W279A (right) to illustrate the gap in the active site created by the disruption of the interaction between W279 and Y410 that assists in active‐site closure and formation of the catalytic active‐site contour. Water is proposed to ingress through this gap and attach at C3 of **2** to generate **8** (PDB ID: 3G4F^9^).

These results establish an essential role for Trp279 in DCS catalysis. In the WT enzyme, residue 279 prevents water access to **5**, which is also stabilised through cation–π interactions with the indole ring. Trp279, hence, facilitates ring closure to **6** and proton loss to generate **7**. Replacement of Trp279 with tyrosine had only a modest effect on the outcome, with a 7 % increase in **8**. However, if Trp279 was replaced with smaller, non‐aromatic residues the quantity of **8** relative to **7** increased in a manner that depended on the van der Waals volume of the residue (Figure 2), irrespective of the hydrophobicity of the amino acid in position 279. Replacement with Gln, Asp or Glu only significantly affected the product distribution and not the catalytic efficiency of the enzymes, thus suggesting that changes to the size of residue 279 might open a channel that allowed water access to the active site of DCS.^21^ The size of the channel appears to depend on the size of residue 279, so that small residues allow for the generation of larger amounts of **8**. Inspection of the X‐ray crystal structure of DCS^9^ reveals the G1/G2 helix break motif first identified by Pandit et al. (Figure 1).^20^ This motif was missed in an earlier report in which a homology model for DCS based on *epi*‐aristolochene synthase was used.^21^ N403 and L405 sit at either end of the helix‐break motif, which suggests that the G1/G2 helix break motif is important for essential loop movements of terpene synthases, including those found in plants.^19^, ^21^ Aside from W279, N403 and L405, the active site of DCS is highly robust to site‐directed mutagenesis, in that changes to G276, I130, T407, C408, G409, L413, E455 and M523—all of which are located in or around the active site—do not lead to the generation of products other than **7**.^26^ This is in stark contrast to many bacterial and fungal terpene synthases for which changes to the active‐site composition often lead to alternative products.^3^, ^27^, ^28^, ^29^, ^30^, ^31^, ^32^, ^33^, ^34^, ^35^, ^36^, ^37^, ^38^ Plant terpene synthases appear to possess a robust architecture that, in most cases, ensures product fidelity. There are, however, a few hot spots where mutation alters the reactivity dramatically and, in the case of DCS, these are found in regions that control the movements of loops involved in closure of the active site prior to formation of the Michaelis complex.^8^, ^9^, ^19^, ^21^ Specifically, in addition to the G1/G2 helix mutants reported previously,^21^ Trp279 mediates a loop movement to ensure that proton loss occurs in the final step of DCS catalysis, as opposed to allowing water to cation **5** to generate **8**. Because W279 is located directly across the active site from Y410, these two residues may form a favourable hydrophobic contact with the substrate as it folds into its reactive conformation with the active site. The aromatic nature of W279 does not appear to determine product distribution because replacement with even negatively charged aspartate or glutamate, or neutral alkyl residues, such as Leu or Ala, do not alter the reaction products; they only increase the proportion of water captured in direct proportion to the van der Waals volume of the side chain. Subsequently, the diphosphate group may act as a general base for the final deprotonation step (Figure 3). If W279 is replaced with a smaller, non‐aromatic residue this process may be perturbed; C1 and C6 of **1** (Scheme 1 and Figure 3) are too far apart to facilitate the final ring closure and extra space in the active site opens a pore,^21^ whereby water can enter the active site and **5** is quenched (Figure 3). It is also notable that alteration of the C terminus through the addition of a hexahistidine tag led to the production of significant quantities of **8**; this is consistent with a precise series of loop movements that effect closure of the active site of DCS. As mentioned above, water molecules have been observed in the crystal structures of several terpene synthases, in both open and closed conformations.^2^, ^7^, ^9^, ^18^ These water molecules, however, do not take part in reactions and simply cushion the substrate in the active site.^23^ It is perhaps surprising that water molecules remain tightly bound in an uncreative state, even in mutant enzymes in which the active site has been altered. In the case of DCS, we have never observed nucleophilic capture of the bicyclic cadinenyl cation (**6**; Scheme 1).

Terpene synthases generate many high‐value products with applications, for instance, as drugs, agrochemicals or fragrances. Understanding the intricate details of their catalytic mechanism will lead to improved methods for the production of naturally occurring terpenes^39^ and help the development of designer products with new or improved properties.^40^, ^41^, ^42^

## Experimental Section

**Introduction of C‐terminal hexahistidine tag into DCS**: The gene encoding WT‐DCS was available in a pET21d vector from previous work.^9^, ^10^ A single nucleotide deletion was required to bring the His_6_ coding sequence of pET21d in frame with the DCS coding sequence. A Quickchange site‐directed mutagenesis kit was used to introduce the desired deletion, according to the manufacturer's instructions. PCR primers were as follows: 5′‐GAACC AATTG CACTT GAGGA TCCGA ATTC‐3′ and 5′‐GAATT CGGAT CCTCA AGTGC AATTG GTTC‐3′. Plasmids were transformed into *E. coli* XL1 Blue and then purified from overnight cultures (lysogeny broth (LB) medium (10 mL) containing ampicillin (100 μg mL^−1^)) by using the miniprep kit, as described by the manufacturer. Deletion was confirmed by DNA sequencing.

**Expression and purification**: DCS‐His_6_ was produced in *E. coli* BL21(DE3) cells that harboured the cDNA for DCS‐His_6_ under control of the T7 promoter. *E. coli* BL21(DE3) cells were gently defrosted on ice before plasmid (1 μL; 60 ng μL^−1^) was added to the cell suspension. The resulting mixture was stored on ice (30 min), heat‐shocked in a water bath (42 °C, 30–35 s) and then returned to the ice (2 min). LB medium (1 mL) was added and the solution was incubated for 1 h at 37 °C with shaking (150 rpm). The cells were harvested by centrifugation (1 min, 3300 *g*), resuspended in a minimum amount of LB medium and spread on an agar plate containing ampicillin (100 μg mL^−1^). The plate was then incubated overnight at 37 °C. A single colony from the agar plate harbouring the transformed cells was used to inoculate LB medium (100 mL) containing ampicillin (100 μg mL^−1^) and the culture was incubated at 37 °C with shaking (150 rpm) overnight. The overnight culture (10 mL) was transferred to each of 6×500 mL of LB medium containing the same concentration of ampicillin as before. Cells were incubated at 37 °C with shaking (150 rpm). When an optical density (OD_600_) of 0.6 was reached, isopropyl β‐d‐1‐thiogalactopyranoside (IPTG) was added (0.5 mm final concentration) and the cultures were incubated for 24 h with shaking (250 rpm), at 20 °C. Cells were harvested by centrifugation at 5 °C (4200 *g*, 10 min). The supernatant solution was discarded and the pellets were stored at −20 °C.

Pellets were allowed to thaw at 5 °C and resuspended in cell lysis buffer (50 mL; 20 mm Tris‐Base, 5 mm β‐mercaptoethanol (βME), pH 8) and stirred gently for 1 h at 0 °C. Cells were then disrupted by sonication at 5 °C (40 % amplitude for 3 min with 5 s on/10 s off cycles) and the resulting suspension was centrifuged at 5 °C (17 000 *g*, 30 min). SDS‐PAGE analysis showed that protein was in the soluble fraction and the pellets were discarded. The supernatant solution was then loaded onto a 2 cm Amintra nitrilotriacetic acid (NTA) Ni^2+^ column (Expedeon, Over, UK) and eluted under gravity‐controlled drip flow. After 40 min, the column was washed with four column volumes (CV) of binding buffer (Tris**⋅**HCl 100 mm, βME 5 mm, NaCl 500 mm, imidazole 5 mm, pH 8). The column was then washed with a gradient of imidazole (from 5 to 300 mm, 20 CV) in binding buffer. DCS‐His_6_ eluted in the range 60–100 mm imidazole; column fractions were analysed by SDS‐PAGE. The fractions containing pure protein corresponding to a molecular weight of 64 000 (DCS‐His_6_) were pooled, dialysed overnight (10 mm Tris‐Base, 5 mm βME, pH 7.5; molecular weight cutoff (MWCO) 30000) and then concentrated to a final volume of about 5 mL (AMICON system, YM 30). The solution was aliquoted and stored at 0 °C. The concentration of protein was estimated by using the method of Bradford.^24^

Site‐directed mutagenesis of recombinant DCS‐His_6_ and mutagenic primers is described in the Supporting Information. The expression and purification of mutant DCS‐His_6_ enzymes was identical to that described for the WT.

**Analytical incubations of DCS‐His_6_ and mutants with 1**: Compound **1** (25 μL, 10 mm) was added to assay buffer (250 μL; 20 mm Tris, 5 mm βME, 10 mm MgCl_2_ at pH 7.5) followed by addition of enzyme (100 μL, 40 μm). The aqueous solution was overlaid with HPLC‐grade pentane (0.5 mL) and the resulting mixture was incubated with gentle agitation (18–24 h) at 25 °C. The incubations were repeated without enzyme as negative controls. The pentane extracts were then analysed by GC‐MS as described in the Supporting Information.

**Steady‐state kinetics of DCS‐His_6_ and mutants**: Kinetic assays were performed according to the standard, linear range, micro‐assay procedure previously developed for DCS (see the Supporting Information).^9^, ^10^

## Supporting information

As a service to our authors and readers, this journal provides supporting information supplied by the authors. Such materials are peer reviewed and may be re‐organized for online delivery, but are not copy‐edited or typeset. Technical support issues arising from supporting information (other than missing files) should be addressed to the authors.

Supplementary
