# Supplementary material for: Nucleophilic Water Capture or Proton Loss: Single Amino Acid Switch Converts δ‐Cadinene Synthase into Germacradien‐4‐ol Synthase
Source: Chembiochem. 2017 Nov 23;19(1):100–5. doi: 10.1002/cbic.201700531 (PMC5814876; doi:10.1002/cbic.201700531)
Supplement: Supplementary file 1 — Supplementary [file CBIC-19-100-s001.pdf]

CHEMBIOCHEM

Supporting Information

Nucleophilic Water Capture or Proton Loss: Single Amino Acid Switch Converts δ-Cadinene Synthase into Germacradien-4-ol Synthase

Marianna Loizzi, Veronica González, David J. Miller, and Rudolf K. Allemann*[a]

cbic_201700531_sm_miscellaneous_information.pdf

Table of Contents

1. **Materials and general methods** **S2**
2. **Introduction of C-terminal 6xHis tag into DCS** **S2**
3. **Production of DCS-His_6** **S3**
4. **Purification of DCS-His_6** **S3**
5. **Site direct mutagenesis** **S4**
6. **Steady-state kinetics parameters of DCS-His_6 and mutants** **S5**
7. **Michaelis-Menten plots for DCS-His_6 and mutants** **S6**
8. **Analytical Incubations of DCS-His_6 and mutants with FDP** **S7**
9. **GC-MS data** **S8**
10. **References** **S10**

1. Materials and general methods. Oligonucleotides and primers for site directed mutagenesis were from Sigma (UK). *Pfu* DNA polymerase and DpnI restriction enzyme were purchased from Fisher (UK). Site directed mutagenesis was carried out using the Quickchange site-directed mutagenesis kit (Stratagene) according to the manufacturer's instructions. QIAGEN miniprep kit was used for the purification of plasmids according to the manufacturer's instructions. All the mutated plasmids were confirmed by DNA sequence analysis using Eurofins DNA sequencing service. Protein expression was induced by the use of isopropyl-β-D-1-thiogalactopyranoside (IPTG). A prestained protein size marker (6.5-175 kDa) was used to identify protein by 10% SDS-PAGE gel. An amicon YM30 membrane was used for protein concentration. Protein concentration was measured by the Bradford method using bovine serum as the calibration standard.[1]

[1-^{3}H-FDP] was purchased by American Radiolabeled Chemicals. Unlabelled FDP was available from previous studies and synthesised following Poulter's two step protocol (chlorination/diphosphorylation)[2,3] from commercially available *trans,trans*-farnesol. [1-^{3}H]-FDP was diluted by addition of unlabelled FDP to give a final specific activity of 75 mCi/mmol.

All other chemicals were from Sigma-Aldrich, Fisher or Melford.

GC-MS analysis of incubation products was performed on a Hewlett Packard 6890 GC apparatus fitted with: column A = J&W Scientific DB-5MS column (30 m x 0.25 mm internal diameter), column B= Agilent J&W DB-35MS (30 m x 0.25 mm internal diameter); and a Micromass GCT Premiere detecting in the range *m/z* 50-800 in the EI$^+$ mode with scanning every 0.95 sec with a scan time of 0.9 s. Method 1: injection port 100 °C; split ratio 5:1; initial pressure 1 kPa; initial temperature 80 °C, ramp of 4 °C/min to 180 °C (2 min hold) flow 1 mL/min; Method 2: injection port 100 °C; split ratio 5:1; initial pressure 1 kPa; initial temperature 80 °C (1 min hold), ramp of 4 °C/min to 180 °C (15 min hold), to 250 °C (20 °C/min, 4 min hold).

2. Introduction of C-terminal hexahistidine tag into DCS. A single nucleotide deletion was required to bring the His_6 coding sequence of pET21d in frame with the DCS coding sequence. A Quickchange site-directed mutagenesis kit was used to introduce the desired deletion according to the manufacturer's instructions. The primers used for the deletion were as follows:

Forwards: 5'-GAACCAATTGCACTTGAGGATCCGAATTC-3'

Reverse: 5'-GAATTCGGATCCTCAAGTGCAATTGGTTC-3'

Plasmids were transformed into *E. coli* XL1 Blue and then purified from overnight cultures (10 mL LB medium containing ampicillin 100 μg/mL) using the miniprep kit as described by the manufacturer. Deletion was confirmed by DNA sequencing.

3. Production of DCS-His$_6$. DCS-His$_6$ was produced in *E. coli* BL21(DE3) cells that harboured the cDNA for DCS-His$_6$ under the control of the T7 promoter. *E. coli* BL21(DE3) cells were gently defrosted on ice before 1 μL of plasmid (60 ng/μL) was added to the cell suspension. The resulting mixture was stored on ice (30 min), heat shocked in a water bath (42 ^{o}C, 30-35 s) and then returned to the ice (2 min). 1 mL of LB medium was added and the solution was incubated for 1 h at 37 ^{o}C while shaking. The cells were harvested by centrifugation (1 min, 3300 *g*), resuspended in a minimum amount of LB medium and spread on an agar plate containing ampicillin (100 μg/ml). The plate was then incubated overnight at 37 °C.

A single colony from the agar plate harbouring the transformed cells was used to inoculate 100 mL of LB medium containing ampicillin (100 μg/mL) and the culture was allowed to grow at 37 °C with shaking (150 rpm) overnight. 10 mL of the overnight culture was transferred to each of 6 x 500 mL of LB medium containing the same concentration of ampicillin as before. Cells were incubated at 37 °C with shaking (150 rpm), when OD_{600} was 0.6, IPTG was added (0.5 mM final concentration) and the cultures were incubated for 24 h with shaking (250 rpm), at 20 °C. Cells were harvested by centrifugation at 5 °C (4200 *g*, 10 min). The supernatant solution was discarded and the pellets were stored at -20 °C.

4. Purification of DCS-His$_6$. Pellets were allowed to thaw at 5 °C and resuspended in 50 mL of cell lysis buffer (20 mM Tris-Base, 5 mM β-mercaptoethanol (βME), pH 8) and stirred gently for 1 h at 0 °C. Cells were then disrupted by sonication at 5 °C (40 % amplitude for 3 min with 5 sec on/10 s off cycles) and the resulting suspension was centrifuged at 5 °C (17000 *g*, 30 min). SDS-PAGE analysis showed that protein was in the soluble fraction and the pellets were discarded. The supernatant solution was then loaded onto a 2 cm Amintra NTA (Nitrilotriacetic acid) Ni^{2+} column (Expedeon^{LTD}) and eluted under gravity controlled drip flow. After 40 minutes the column was washed with 4 column volumes (CV) of binding buffer (Tris-HCl 100 mM, βME 5 mM, NaCl 500 mM, imidazole 5 mM, pH 8). The column was then washed with a gradient of imidazole (from 5 to 300 Mm, 20 CV) in binding buffer. DCS-His$_6$ eluted in the range 60-100 mM imidazole, column fractions were analysed by SDS-PAGE. The fractions containing pure protein corresponding to a molecular weight of 64000

(DCS-His$_6$) were pooled, dialysed overnight (10 mM Tris-Base, 5 mM βME, pH 7.5 - MWCO 30000) and then concentrated to a final volume of ~ 5 mL (AMICON system, YM 30). The solution was aliquoted and stored at 0 °C. The concentration of protein was estimated using the method of Bradford.[1]

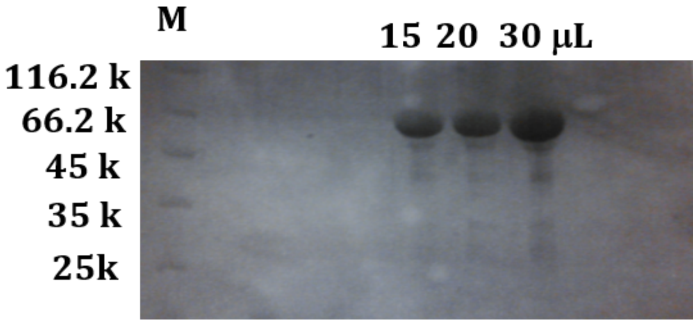

Figure S1. 10% SDS-polyacrylamide gel of pure concentrated DCS-His$_6$. (M): protein marker; (15): 15 µL; (20) 20 µL and (30) 30 µL of purified DCS-His$_6$.

5. Site direct mutagenesis Site directed mutagenesis was carried out using the Quickchange site-directed mutagenesis kit (Stratagene) according to the manufacturer's instructions. QIAGEN miniprep kit was used for the purification of plasmids according to the manufacturer's instructions. All the mutated plasmids were confirmed by DNA sequence analysis.

The mutagenic primers for DCS were as follow:

**W279Q**	Forward: 5' GAGTTGTTGAAGGTTACTTTCAGATCTCTGGAGTGTACTTTG 3'<br>Reverse: 5' CAAAGTACACTCCAGAGATCTGAAAGTAACCTTCAACAACTC 3'
**W279D**	Forward: 5' GTTGTTGAAGGTTACTTTGACATCTCTGGAGTGTACTTTG 3'<br>Reverse: 5' CAAAGTACACTCCAGAGATGTCAAAGTAACCTTCAACAAC 3'
**W279M**	Forward: 5' GAGTTGTTGAAGGTTACTTTATGATCTCTGGAGTGTACTTTG 3'<br>Reverse: 5' CAAAGTACACTCCAGAGATCATAAAGTAACCTTCAACAACTC 3'
**W279L**	Forward: 5' GAGTTGTTGAAGGTTACTTTCTGATCTCTGGAGTGTACTTTG 3<br>Reverse: 5' CAAAGTACACTCCAGAGATCAGAAAGTAACCTTCAACAACTC 3'
**W279A**	Forward: 5' GAGTTGTTGAAGGTTACTTTGCGATCTCTGGAGTGTACTTTG 3'<br>Reverse: 5' CAAAGTACACTCCAGAGATCGCAAAGTAACCTTCAACAACTC 3
**W279Y**	Forward: 5' GAGTTGTTGAAGGTTACTTTTACATCTCTGGAGTGTACTTTGAG 3'<br>Reverse: 5' CTCAAAGTACACTCCAGAGATGTAAAAGTAACCTTCAACAACTC 3'

PCR procedure

Sterile H_2O (39 µL), Pfu-polymerase buffer (5 µL), dNTPs (1 µL, 10 mM), 2 µL of forward and reverse primers 0.1 mM, 1 µL template DNA 0.01 pmol and 1 µL Pfu-polymerase (2.5 U/µL) were added to a PCR tube and followed PCR protocol (Table 1). The parental DNA was digested with 1 µL *Dpn*I (10 U/µL) for 1 h at 37 °C. The reaction was cooled on ice before transformation into XL1-Blue *E. coli*

cells. Mutations were confirmed by DNA sequence analysis using Eurofins MWG Operon's DNA sequencing service.

Table S1. PCR protocol

Step	Temperature (°C)	Time (min)
Initial denaturation	95	3
Denaturation<br>Annealing<br>Elongation (x 15)	95<br>55<br>72	1<br>2<br>12
Final elongation	72	10

6. Steady-state kinetics parameters of DCS-His$_6$ and mutants. Kinetics assays were carried out according to the standard, linear range, micro-assay procedure previously developed for DCS.[4] This protocol involved the incubation of varying amounts of [1-^{3}H]-FDP (specific activity 75 mCi/mmol), at pH 7.5 with fixed concentrations of purified DCS-His$_6$ (or mutant) (100 nM) in 20 mM Tris buffer containing 1 mM DTT, 5mM $MgCl_2$, pH 7.5. The reaction mixtures containing buffer, FDP and protein were prepared on ice in a total volume of 250 µL and were overlaid with ca 0.8 mL of HPLC-grade hexane prior to incubation. The assay mixtures were incubated at room temp (30 °C) for 10 min. The reactions were immediately ice-cooled and quenched by addition of 200 µL of 100 mM EDTA (pH 8.5) and brief vortexing. The hexane overlay and two additional 1 mL hexane extracts were passed through a short pipette column containing silica gel. The column was washed with additional hexane (1 mL) and the combined filtrates were analysed by liquid scintillation counting using 15 mL of scintillation cocktail Ecoscint O. For the mutants producing the alcohol GD4OL, a solution of hexane:diethylether 11:1 was used instead of pure hexane. Steady-State kinetic parameters for DCS-His$_6$ and mutants were obtained by direct fitting of the data to the Michaelis-Menten equation by nonlinear least squares regression in conjunction with the graphical procedures developed by Lineweaver-Burk using the commercial SigmaPlot package (Systat Software).

7. Michaelis-Menten plots for DCS-His$_6$ and mutants.

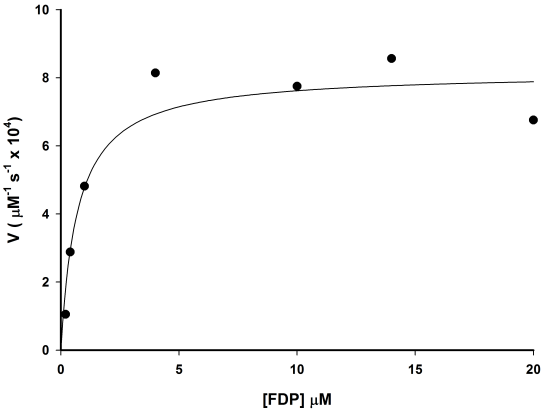

Figure S1. Representative Michaelis-Menten plot for the production of radiolabelled products by DCS-His$_6$.

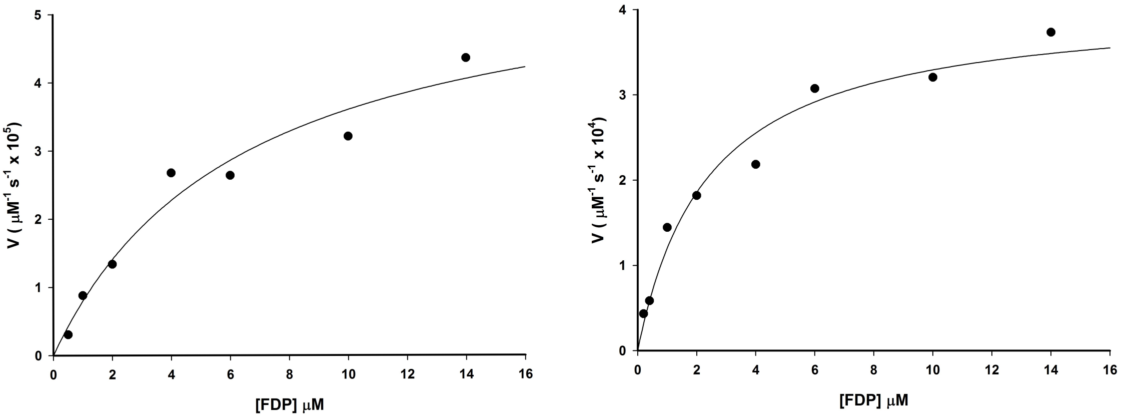

Figure S2. Representative Michaelis-Menten plots for the production of radiolabelled products by DCS-W279D (left) and (right) DCS-W279A.

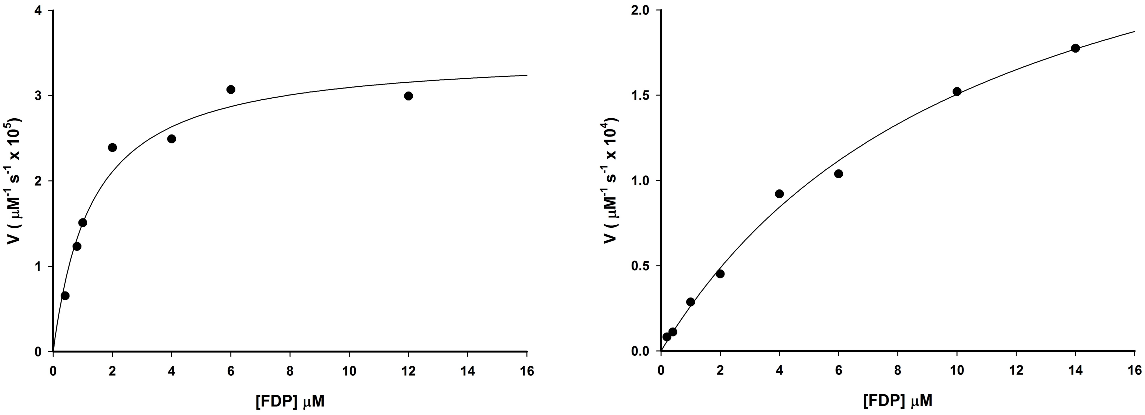

Figure S3. Representative Michaelis-Menten plots for the production of radiolabelled products by DCS-W279E (left) and DCS-W279L (right).

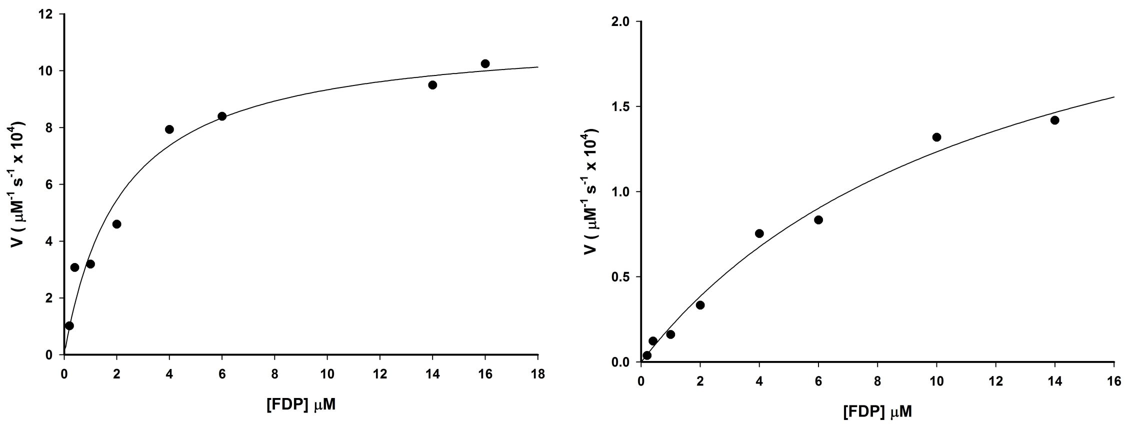

Figure S4. Representative Michaelis-Menten plots for the production of radiolabelled products by DCS-W279M (left) and DCS-W279Q (right).

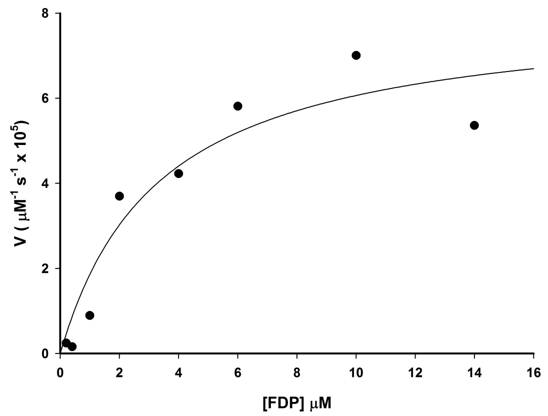

Figure S5. Representative Michaelis-Menten plots for the production of radiolabelled products by W279Y-DCS.

8. Analytical Incubations of DCS-His$_6$ and mutants with FDP. FDP (25 µL, 10 mM) was added to assay buffer (250 µL, 20 mM Tris, 5 mM βME, 10 mM $MgCl_2$ at pH 7.5) followed by addition of enzyme (100 µL, 40 µM). The aqueous solution was overlaid with HPLC grade pentane (0.5 mL) and the resulting mixture was incubated (18-24 h) at 25 °C. The incubations were repeated without enzyme as negative controls. The pentane extracts were then analysed by gas chromatography-mass spectrometry (GC-MS) as described in Materials and general methods (Section 1).

9. GC-MS data

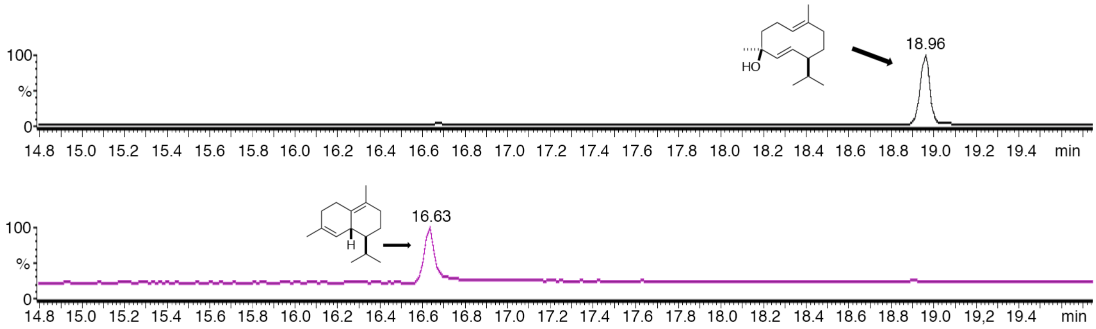

Figure S7. Total ion chromatograms of the pentane extractable products arising from incubation of FDP with germacradien-4-ol synthase (top, method 1, column B) and DCS-His$_6$ (bottom, method 1, column B).

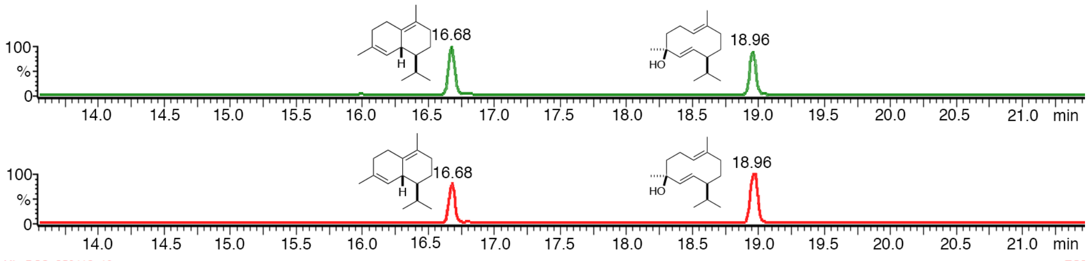

Figure S8 Total ion chromatograms of the pentane extractable products arising from incubation of DCS-W279E (top) and DCS-W279D (bottom) with FDP. Method 1, column B.

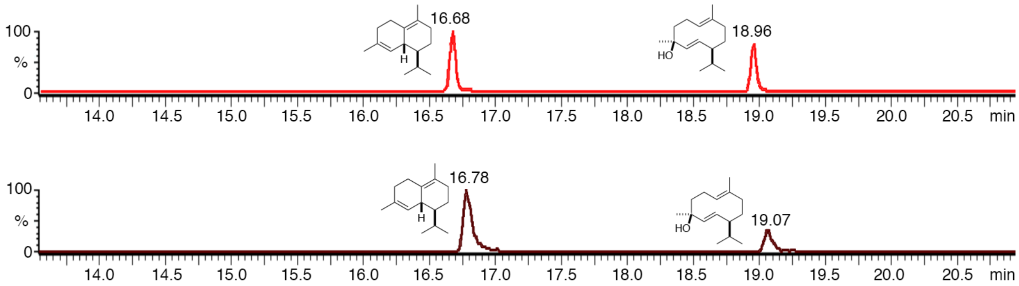

Figure S9. Total ion chromatograms of the pentane extractable products arising from incubation of W279Q (top, method 1, column B) and W279L (bottom, method 2, column B) with FDP.

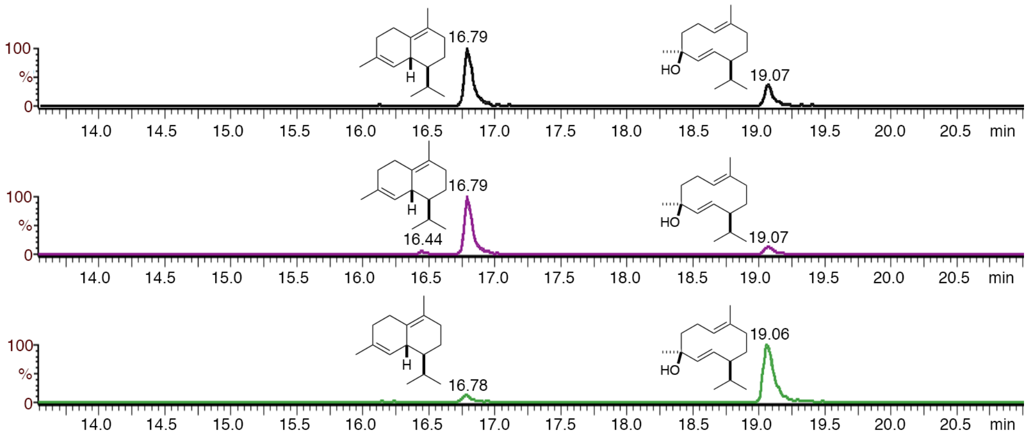

Figure S10. Total ion chromatograms of the pentane extractable products arising from incubation of W279M (top), W279Y (middle) and W279A (bottom) with FDP. Method 2, column B

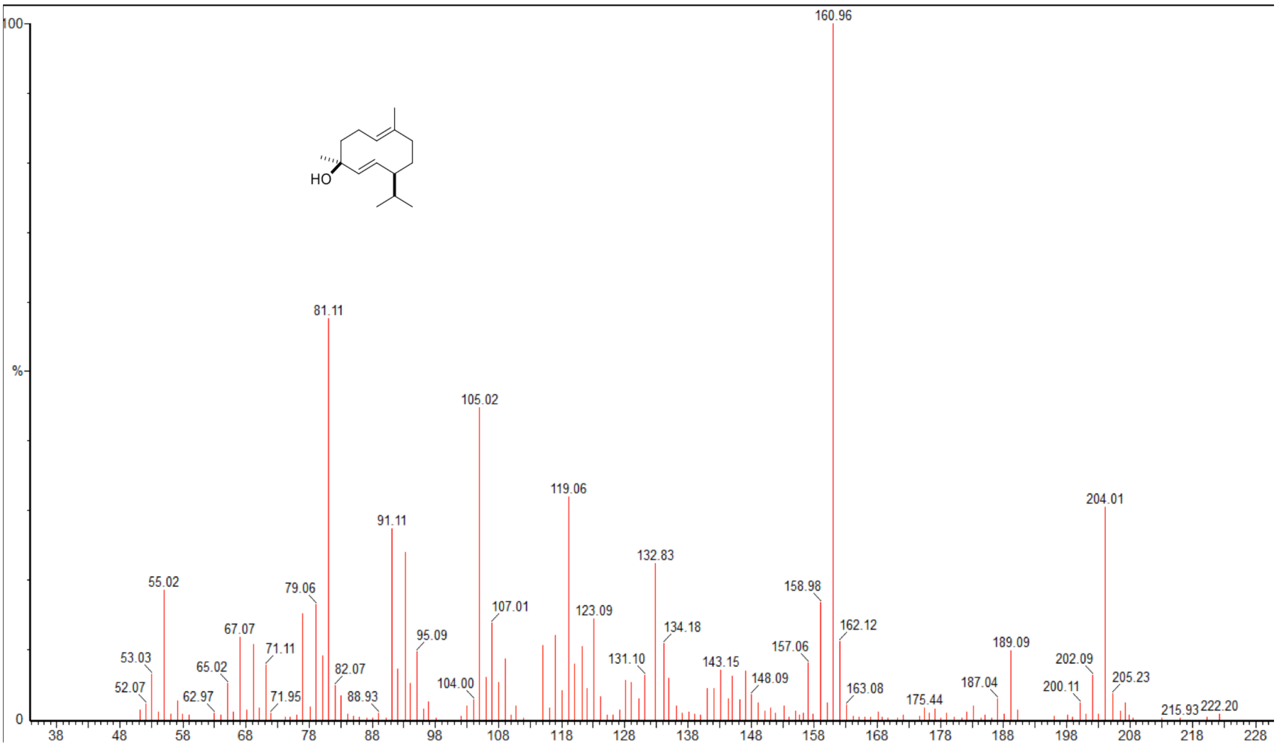

Figure S1. Mass spectrum of the compound eluting at 18.96 min. germacradien-4-ol.

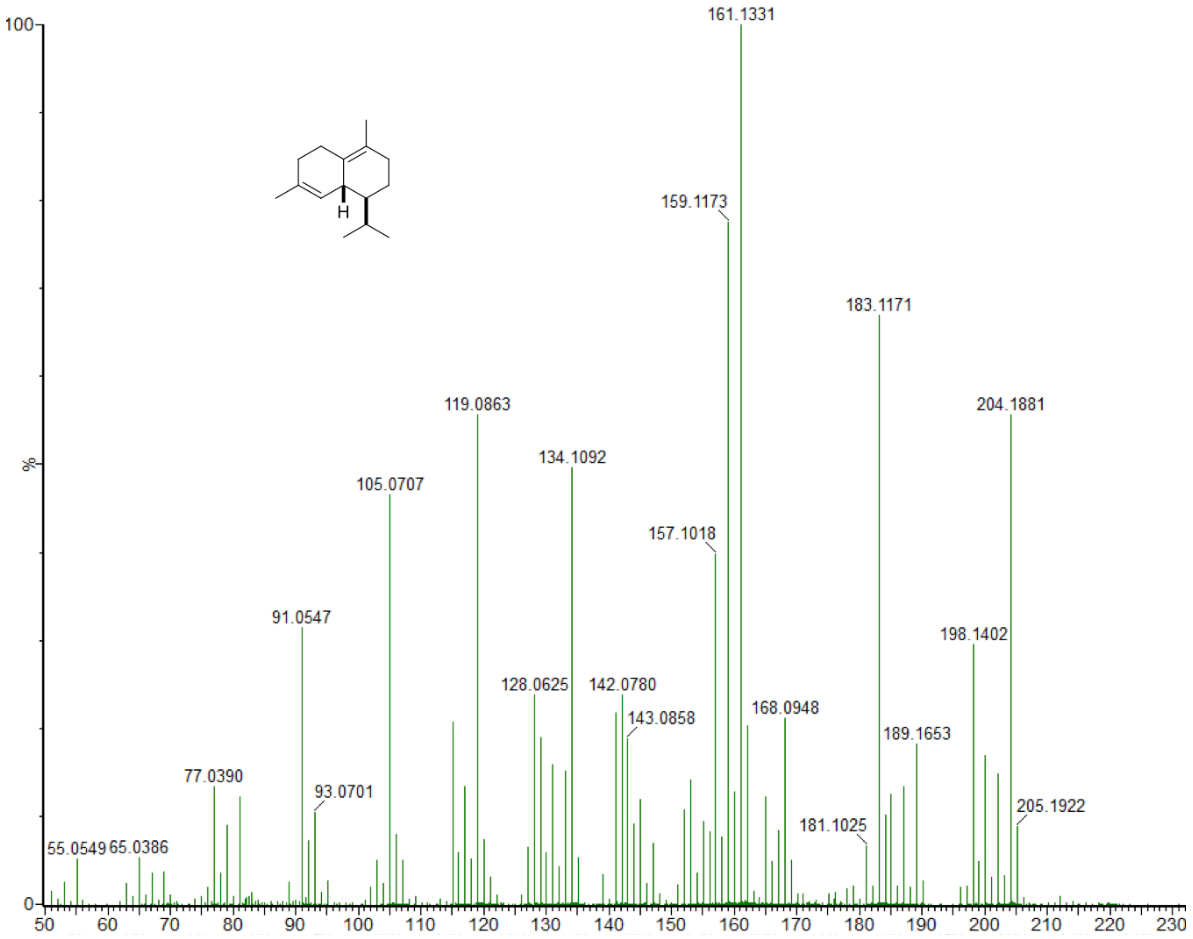

Figure S2. Mass spectrum of the compound eluting at 16.63 min. δ-cadinene.

10. References

[1] M. M. Bradford, *Anal. Biochem.* **1976**, *72*, 248–254.

[2] V. J. Davisson, A. B. Woodside, T. R. Neal, K. E. Stremler, M. Muehlbacher, C. D. Poulter, *J. Org. Chem*. **1986**, *51*, 4768-4779.

[3] Woodside, A. B.; Huang, Z.; Poulter C. D. *Org. Synth. Coll. Vol.* **1993**, *8*, 616-620.

[4] J. A. Faraldos, D. J. Miller, V. González, Z. Yoosuf-Aly, O. Cascón, A. Li, R. K. Allemann, *J. Am. Chem. Soc.* **2012**, *134*, 5900–5908.
